# Systematic Screening for Deep Vein Thrombosis in Critically Ill Inpatients With COVID-19: Impact on the Incidence of Venous Thromboembolism

**DOI:** 10.3389/fmed.2020.624808

**Published:** 2021-01-14

**Authors:** François-Xavier Lapébie, Vincent Minville, Agnès Ribes, Bertrand Combis, Arthur Thery, Thomas Geeraerts, Stein Silva, Alessandra Bura-Rivière, Fanny Vardon-Bounes

**Affiliations:** ^1^Department of Vascular Medicine, Toulouse University Hospital, Toulouse, France; ^2^UMR 1027 INSERM, Toulouse III – Paul Sabatier University, Toulouse, France; ^3^Department of Anesthesiology and Critical Care, Toulouse University Hospital, Toulouse, France; ^4^UMR 1048 INSERM, I2MC, Toulouse III – Paul Sabatier University, Toulouse, France; ^5^Laboratory of Hematology, Toulouse University Hospital, Toulouse, France; ^6^ToNIC (Toulouse Neuro-Imaging Center) INSERM, Toulouse III – Paul Sabatier University, Toulouse, France; ^7^UMR 1031 INSERM, StromaLab, Toulouse III – Paul Sabatier University, Toulouse, France

**Keywords:** COVID-19, venous thromboembolism, ultrasonography, deep vein thrombosis, intensive care unit

## Abstract

**Background:** Several studies suggest an increased incidence of thrombosis in COVID-19 patients. However, evidence on how to prevent and even treat it is scarce. The aim of this study was to compare the cumulative incidence of venous thromboembolism (VTE) of two different methods for lower extremity deep vein thrombosis (LE-DVT) diagnosis: systematic vs. clinically guided complete compression venous ultrasonography (CCUS). We conducted a monocentric, prospective, open-label, non-randomized study. All consecutive patients admitted in three intensive care units (ICUs) of University Hospital of Toulouse for COVID-19 pneumonia were included: one performed systematic screening for LE-DVT, the others did not. The primary outcome was the 21-day cumulative incidence of VTE. The secondary end points were the 21-day cumulative incidences of major bleeding and death.

**Results:** Among the 78 patients included, 27 (34.6%) underwent systematic screening for DVT 7 ± 2 days after ICU admission. Thirty-two patients (41.0%) were diagnosed with VTE, with a 21-day cumulative incidence of 42.3% (95% CI, 31.4–55.2), without difference between screened and non-screened patients (hazard ratio 1.45, 95% CI, 0.72–2.93). In the screened group, the frequency of isolated DVT was higher (25.9 vs. 5.9%, *p*-value = 0.027), but the frequency of pulmonary embolism was not reduced (25.9 vs. 29.4%, *p*-value = 0.745). The 21-day cumulative incidences of major bleeding and death were 9.6% (95% CI, 4.7–19.2) and 10.3% (95% CI, 5.0–20.8), respectively, without difference between the two groups.

**Conclusions:** A systematic screening for DVT in patients hospitalized in ICU was not associated with a higher diagnosis of VTE or a reduced diagnosis of PE.

## Introduction

Critically ill patients are at high risk for developing venous thromboembolism (VTE), with a frequency ranging from 5.1 to 15.5% despite the use of low-molecular-weight heparin (LMWH) thromboprophylaxis (1). Incidence is particularly high in patients diagnosed with sepsis, with a 30-day cumulative incidence of VTE of 12.5%, and 31% in patients with acute respiratory distress syndrome (ARDS) (2).

Early data suggested increased incidence of thrombosis in COVID-19 patients, particularly in critically ill patients, and have been confirmed since. In a multicentric study of 184 patients hospitalized in intensive care unit (ICU) with COVID-19 pneumonia, the adjusted cumulative incidence of thrombotic complication was 49% [95% confidence interval (95% CI), 41–57], with a majority of pulmonary embolism (PE) (3). In another monocentric study of 107 ICU patients with COVID-19, the cumulative incidence of PE at 15 days of admission was 20.4% (95% CI, 13.1–28.7), and frequency of PE was twice as high as that of influenza ICU patients admitted the year before (4). In a propensity score matching analysis, COVID-19 patients with ARDS developed more PE than non-COVID-19 patients with ARDS, odds ratio (OR) 6.2 (95% CI, 1.6–23.4) (5).

In a systematic overview of 80 consecutive autopsies of the COVID-19 deaths, 17 PE (21%) were found, of whom eight were fatal. In each of these deaths as well as in 15 others (32 cases, 40%), lower extremities deep vein thrombosis (LE-DVT) were found. The most frequent cause of death was pneumonia, followed by PE combined with pneumonia (6).

The clinical history and physical examination are of poor utility in determining the probability and risk of deep vein thrombosis (DVT) in the ICU (7, 8). An ultrasound-based DVT screening may be helpful to identify early LE-DVT and therefore adapt treatment in order to prevent progression to PE.

## Method

### Aim of the Study

The aim of the study was to determine the impact of routine screening for DVT on the number of cumulative VTE at day 21 on patients admitted in ICU for COVID-19 pneumonia. The secondary end points were the cumulative incidences of major bleeding and death at day 21.

### Study Design and Ethics

This prospective, monocentric, cohort study (ECHO-VID) was conducted in Toulouse University Hospital from March 10, 2020, to May 7, 2020. The management of the patients was not modified during the study, since in the site performing systematic screening for LE-DVT, the ultrasound screening is easily accessible and done, while in the other two centers, it is not. Patients were informed that their data would be used for the study. The local ethic committee gave its consent to the collection of the data and the study is declared in the register of observational studies of Toulouse University Hospital (number's register: 2020-091).

### Patients

Consecutive patients admitted in three medico-surgical ICUs for COVID-19 ARDS were identified. SARS-CoV-2 pneumonia was confirmed by a reverse transcription polymerase chain reaction (RT-PCR) test on a nose/throat swab or sputum sample positive for SARS-CoV-2, or, in patients with a negative RT-PCR but with symptoms consistent with COVID-19, by abnormalities highly suspicious of COVID-19 on a chest computed tomography (CT) scan in the absence of an alternative diagnosis. Patients were excluded if their length of ICU stay was <72 h or if acute VTE was already present at ICU admission.

### Procedures

The three ICUs follow the same standardized procedures and clinical protocols. Thrombosis prophylaxis was systematically given in COVID-19 patients without major risk of bleeding. Of the three ICUs, one benefitted from the passage of vascular physicians for systematic bedside lower extremities complete compression venous ultrasonography (LE-CCUS screened group) at day 7 ± 2. The other units did not perform systematic screening (non-screened group). All the centers performed CCUS in cases of suspected DVT (localized tenderness, pitting oedema or swelling in each lower extremity, and central venous catheter dysfunction). Proximal DVT was defined as the thrombus involving at least the popliteal vein and above, and distal DVT was defined as the thrombus involving veins below the popliteal level. Isolated DVT was defined as DVT without associated PE. CT pulmonary angiography (CTPA) was performed only for patients with clinical suspected PE (acute degradation of hemodynamic or respiratory status, difficulties to discontinue mechanical ventilation). The three ICUs strictly applied the same thromboprophylaxis protocol: until April 3, a standard dose anticoagulant thromboprophylaxis with enoxaparin 4,000 IU once-daily or 4,000 IU twice-daily for patients with a body mass index >40 kg/m^2^. From April 3 onwards, patients received an intermediate-dose anticoagulant thromboprophylaxis with enoxaparin 80 IU/kg per day, in one injection for patients with a body weight of ≤100 kg and in two injections for patients with a body weight of >100 kg. In case of severe renal impairment, patients received subcutaneous UFH 5,000 IU two or three times a day without anti-factor Xa monitoring. Patients with acute PE or DVT were treated with therapeutic anticoagulation by intravenous UFH or LMWH (enoxaparin 100 IU/kg twice daily or tinzaparin 175 IU/kg once daily). Patients with indication for oral therapeutic anticoagulation before hospital admission like atrial fibrillation, history of VTE, or mechanical heart valves were switched to enoxaparin 100 IU/kg twice daily, reduced to 100 IU/kg once daily if estimated glomerular filtration rate (eGFR) is between 15 and 29 ml/min/1.73 m^2^, or intravenous UFH with a goal of 0.3–0.6 IU/ml of antifactor Xa activity if GFR ≤ 14 ml/min/1.73 m^2^.

### End Points

The primary outcome was the cumulative incidence of objectively confirmed VTE (symptomatic or not), including PE, LE-DVT, and catheter-associated upper extremity (UE)-DVT, during 21 days in the hospital. The secondary outcomes were the cumulative incidences of major bleeding [International Society of Thrombosis and Haemostasis (ISTH)-defined] and death, during the first 21 days following ICU admission (9). We did not adjudicate deaths to identify fatal PE, as all but one death was due to hypoxemic respiratory failure that can be indistinguishable from fatal PE.

### Statistical Analysis

Categorical variables are presented as numbers and percentages. Continuous variables are presented as means and standard deviations (SD). Comparisons were made using χ^2^ test for categorical variables or Fisher exact test when appropriate, while Student *t*-test and Mann–Whitney test were used for continuous variables. The Kaplan–Meier method was used to estimate cumulative incidence of events, with 95% CI. The association between routine screening using LE-CCUS and VTE diagnosis, major bleeding, and death was analyzed by unadjusted Cox proportional-hazard models, after checking for the proportional-hazard assumption. A *p*-value <0.05 was considered to be statistically significant. Analyses were performed on STATA Statistical software (release 14.2, StataCorp LLC®).

## Results

Between March 10 and May 7, 2020, 85 patients were eligible for the study. One patient was excluded due to PE prior to ICU admission, and six patients were excluded because of ICU length of stay <72 h; one of them required extracorporeal membrane oxygenation (ECMO) and underwent a fatal intracranial bleeding 24 h after VTE diagnosis. Finally, 78 patients were included in the analysis. Twenty-seven (34.6%) received systematic screening for DVT by bedside LE-CCUS 7 ± 2 days after ICU admission. The clinical and biological characteristics of the population are presented in Table 1. Fifty-five patients underwent CTPA: 22 (81.5%) of the LE-CCUS screened group and 33 (64.7%) of the non-screened group, *p*-value = 0.122.

**Table 1:** Characteristics of patients in ICU according to systematic screening or not for LE-DVT.

	**All patients** **[*n* = 78 (%)]**	**LE-CCUS screened group** **[*n* = 27 (%)]**	**Non-screened group** **[*n* = 51 (%)]**	***p-*value**
**Demographic data**				
Male sex	67/78 (85.9)	24/27 (88.9)	43/51 (84.3)	0.739
Age (years)	63.3 ± 13.9	61.5 ± 12.4	64.2 ± 14.6	0.411
BMI (kg/m^2^)	27.7 ± 4.4	28.8 ± 4.0	27.1 ± 4.5	0.107
Days between hospitalization and ICU admission	1.7 ± 2.2	2.0 ± 2.3	1.5 ± 2.2	0.287
SOFA score	5.9 ± 2.2	6.0 ± 2.4	5.9 ± 2.1	0.855
PaO_2_/FiO_2_ ratio	163 ± 62	167 ± 58	160 ± 64	0.617
**Medical history**				
History of VTE	6/78 (7.7)	3/27 (11.1)	3/51 (5.9)	0.412
Surgery during the 3 months before	1/78 (1.3)	0/27 (0.0)	1/51 (2.0)	1.000
Confined to bed in hospital during the 3 months before	2/78 (2.6)	0/27 (0.0)	2/51 (3.9)	0.541
Active cancer	6/78 (7.7)	3/27 (11.1)	3/51 (5.9)	0.412
**Biologic data at ICU admission**				
eGFR (ml/min/1.73 m^2^)	83 ± 27	82 ± 31	83 ± 24	0.811
Hemoglobin (g/dl)	13.0 ± 2.0	12.6 ± 2.0	13.2 ± 2.0	0.178
Leukocytes (G/L)	7.98 ± 3.44	8.53 ± 3.26	7.68 ± 3.53	0.302
Platelets (G/L)	223 ± 98	229 ± 113	220 ± 91	0.703
D-dimer (mg/L)	1.78 ± 1.07	1.88 ± 1.11	1.69 ± 1.05	0.606
Prothrombin (% of activity)	88 ± 12	86 ± 12	89 ± 12	0.445
aPTT ratio	1.15 ± 0.29	1.22 ± 0.56	1.13 ± 0.11	0.176
Fibrinogen (g/L)	6.8 ± 1.4	7.1 ± 2.0	6.7 ± 1.0	0.353
CRP (mg/L)	137.5 ± 90.0	149.0 ± 93.4	131.8 ± 88.8	0.469
**Characteristics of ICU stay**				
Prophylactic anticoagulation at ICU admission	69/78 (88.5)	21/27 (77.8)	48/51 (94.1)	0.057
Therapeutic anticoagulation at ICU admission	7/78 (9.0)	5/27 (18.5)	2/51 (3.9)	0.045
Catecholamine support	56/78 (71.8)	20/27 (74.1)	36/51 (70.6)	0.745
Mechanical ventilation	66/78 (84.6)	25/27 (92.6)	41/51 (80.4)	0.200
RRT	5/78 (6.4)	3/27 (11.1)	2/51 (3.9)	0.334
ECMO	5/78 (6.4)	3/24 (11.1)	2/51 (3.9)	0.334
ICU length of stay (days)	21 ± 15	25 ± 16	18 ± 15	0.053
Hospital length of stay since ICU admission (days)	31 ± 20	36 ± 19	29 ± 20	0.117

*aPPT, activated partial thromboplastin time; BMI, body mass index; CCUS, complete compression venous ultrasonography; CRP, C-reactive protein; DVT, deep vein thrombosis; ECMO, extracorporeal membrane oxygenation; eGFR, estimated glomerular filtration rate; ICU, intensive care unit; LE, lower extremity; PaO_2_/FiO_2_ ratio, ratio of arterial oxygen partial pressure to fractional inspired oxygen; RRT, renal replacement therapy; SOFA, sequential organ failure assessment. Results are expressed as numbers and percentages [n (%)] or mean ± standard deviation*.

There were no difference concerning past medical history between the two groups, except for history of transplantation being more frequent in the LE-CCUS screened group (14.8 vs. 2%, *p*-value = 0.046). Of the total population, six patients (7.7%) had a history of VTE, five (6.4%) had atrial fibrillation, two (2.6%) had chronic heart failure, nine (11.5%) suffered from chronic obstructive pulmonary disease (COPD), and six (7.7%) suffered from active neoplasia. Nine (11.5%) were current smokers, 18 (23.1%) had diabetes mellitus, 41 (53.6%) had hypertension, and 17 (21.8%) had dyslipidemia. There was no difference between the two groups regarding ICU organ support therapy, but therapeutic anticoagulation started at ICU admission was more frequent in the LE-CCUS screened group.

**Figure 1:**
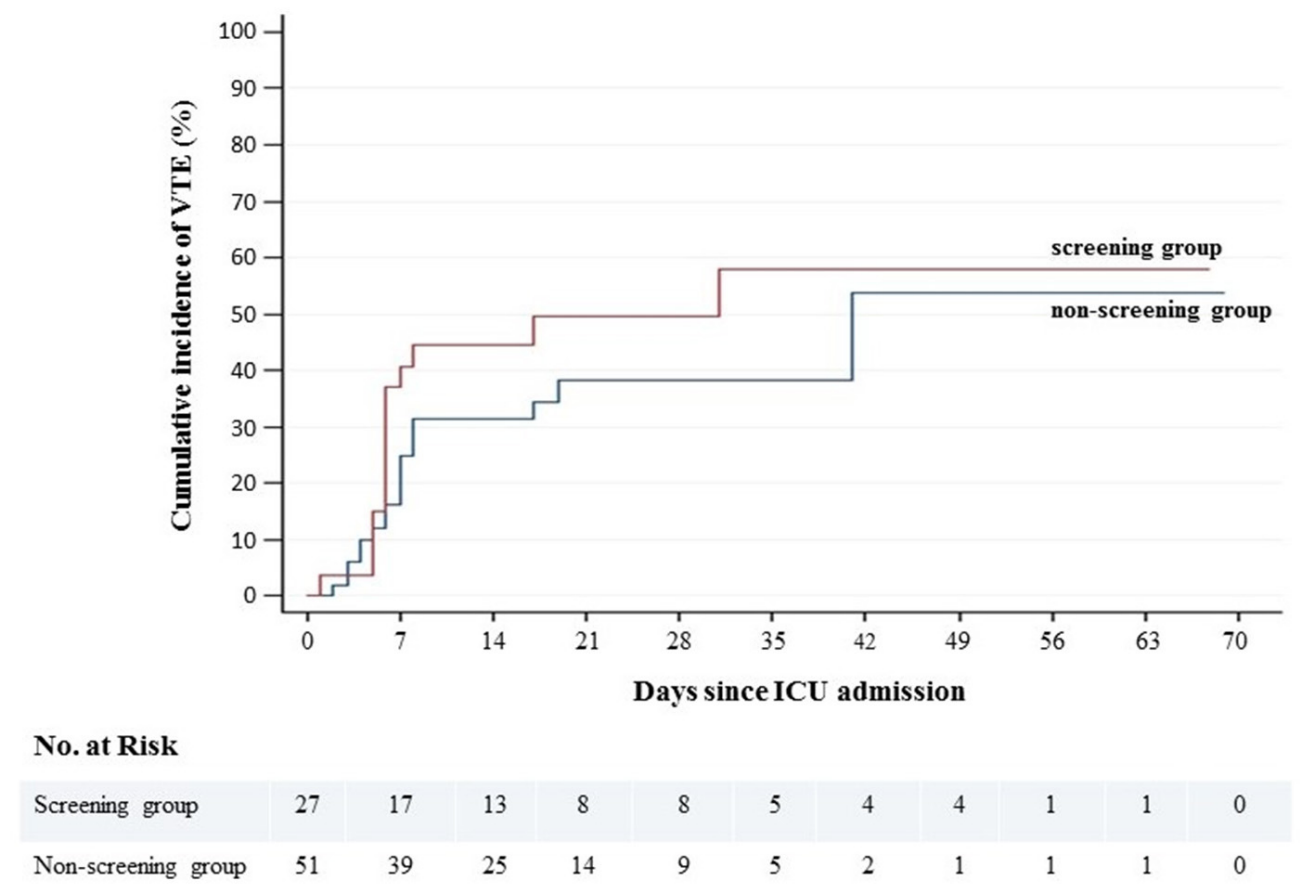
Cumulative incidence of venous thromboembolism according to screening or not for LE-DVT.

During a mean follow-up of 31 ± 20 days, 32 patients (41.0%) were diagnosed with VTE, with a 21-day cumulative incidence of 42.3% (95% CI, 31.4–55.2). There was no difference in VTE cumulative incidence between LE-CCUS screened and non-screened groups (Figure 1, Table 2). Of note, CCUS was performed in six patients (11.8%) in the non-screened group because of suspected DVT. The type of VTE was PE with or without DVT in 22 patients (28.2%), of whom three were subsegmental PE, proximal LE-DVT in one patient (1.3%), isolated distal LE-DVT in six patients (7.7%), catheter-associated UE-DVT in two patients (2.6%), and catheter-associated UE-DVT + distal LE-DVT in 1 patient (1.3%) (Table 3). All but one VTE were diagnosed in patients receiving prophylactic or therapeutic anticoagulation. In the LE-CCUS screened group, the frequency of PE was not reduced (25.9 vs. 29.4%, *p*-value = 0.745), but the frequency of isolated DVT was higher (25.9 vs. 5.9%, *p*-value = 0.027); however, it was mainly isolated distal LE-DVT. After VTE diagnosis, all but one patient received therapeutic anticoagulation, by LMWH or UFH.

**Table 2:** Cumulative incidence of events at 21 days according to systematic screening or not for LE-DVT, and comparison by unadjusted Cox models, with non-screened group as reference.

	**All** **[% (95% CI)]**	**LE-CCUS screened group** **[% (95% CI)]**	**Non-screened group** **[% (95% CI)]**	**Hazard ratio (95% CI)**	***p*-value**
Venous thromboembolism	42.3 (31.4–55.2)	49.5 (32.2–69.9)	38.3 (25.3–55.1)	1.45 (0.72–2.93)	0.296
Major bleeding	9.6 (4.7–19.2)	14.8 (5.8–34.8)	6.9 (2.3–20.0)	1.05 (0.36–3.09)	0.924
Death	10.3 (5.0–20.8)	3.7 (0.5–23.5)	14.0 (6.4–29.2)	0.26 (0.03–2.10)	0.206

*CCUS, complete compression venous ultrasonography; DVT, deep vein thrombosis; LE, lower extremity*.

**Table 3:** Clinical outcomes of patients according to systematic screening or not for LE-DVT.

	**All** **[*n* = 78 (%)]**	**LE-CCUS screened group** **[*n* = 27 (%)]**	**Non-screened group** **[*n* = 51 (%)]**	***p*-value**
Venous thromboembolism	32/78 (41.0)	14/27 (51.9)	18/51 (35.3)	0.157
Pulmonary embolism	22/78 (28.2)	7/27 (25.9)	15/51 (29.4)	0.745
Associated with LE-DVT	9/78 (11.5)	7/27 (25.9)	2/51 (3.9)	0.007
Without LE-DVT	13/78 (16.7)	0/27 (0.0)	13/51 (25.5)	0.003
Isolated DVT	10/78 (12.8)	7/27 (25.9)	3/51 (5.9)	0.027
**Deep vein thrombosis localization**				
Proximal LE-DVT	3/78 (3.9)	3/27 (11.1)	0/51 (0.0)	0.038
Isolated distal LE-DVT	14/78 (18.0)	11/27 (40.7)	3/51 (5.9)	<0.001
Catheter-related UE-DVT	3/78 (3.9)	1/27 (3.7)	2/51 (3.9)	1.000
Major bleeding	14/78 (18.0)	6/27 (22.2)	8/51 (15.7)	0.541
Death	8/78 (10.3)	1/27 (3.7)	7/51 (13.7)	0.250

*CCUS, complete compression venous ultrasonography; DVT, deep vein thrombosis; LE, lower extremity; UE, upper extremity. Results are expressed as numbers and percentages [n (%)] or mean ± standard deviation*.

There was no difference in the cumulative incidence of major bleeding and death between LE-CCUS screened and non-screened groups (Table 2). All but one major bleeding were diagnosed in patients receiving therapeutic or prophylactic anticoagulation, with five ear–nose–throat bleeding, four gastrointestinal bleeding, and two retroperitoneal bleeding. The cause of death was refractory hypoxemia in seven patients (9.0%) and fatal intracranial bleeding in one patient (1.3%) treated by therapeutic dose of UFH for PE.

No statistically significant difference between intermediate-dose and standard dose anticoagulant thromboprophylaxis with enoxaparin was observed for VTE, major bleeding, and death, respective unadjusted HR 0.85 (95% CI, 0.34–2.11), *p*-value = 0.731; 0.66 (95% CI, 0.14–3.09), *p*-value = 0.594; and 0.52 (95% CI, 0.06–4.40), *p*-value = 0.551.

## Discussion

Our study confirms the high cumulative incidence of VTE in critically ill patients with COVID-19 pneumonia (42.3%, 95% CI, 31.4–55.2) despite a prophylactic anticoagulation. Previous studies reported that VTE frequency ranged from 6.6 to 37.0% in comparable critically ill patients, despite thromboprophylaxis and without VTE screening, with a 15-day cumulative incidence that ranged from 20.4 to 27% (3, 4, 10–13). The diagnosis was often made within the first week after ICU admission, with a median time from ICU admission of 6 days in the study of Poissy et al. and a median time from hospital admission of 24 h in the study of Lodigiani et al. (4, 12). Frequency of VTE is higher in case of LE-DVT screening by CCUS in ICU, up to 69%, with DVT ranging from 14.7 to 85.4% but mainly distal (14–17). In the only other study evaluating bilateral leg ultrasound screening, the 21-day cumulative incidences of VTE were 59% (95% CI, 42–72) with a screening approach for LE-DVT and 34% (95% CI, 21–46) when excluding asymptomatic events detected by LE-CCUS (18). Isolated distal DVT are frequent and represent 30–50% of all LE-DVT diagnosed on CCUS series (19–21). Therapeutic anticoagulation is not mandatory if the risk of recurrence is low (22).

To date, routine ultrasound screening for the detection of asymptomatic LE-DVT in COVID-19 patients is not recommended (23, 24). However, to the best of our knowledge, our study is the first to compare LE-CCUS screened and non-screened groups, with no difference found regarding cumulative incidences of VTE, major bleeding, and death. Because the majority of PE originated in the deep venous system of LE, undiagnosed DVT and resultant PE may be an important contributor to hypoxic pulmonary vasoconstriction that would lead to pulmonary hypertension and right ventricular failure in COVID-19 patients, in addition to worsening of ARDS (25). However, in contrast with the relatively frequent report of PE in hospitalized COVID-19 patients, LE-DVT, especially proximal, might be less common and some authors hypothesize that the observed pulmonary vessel occlusions are caused by local thrombi in pulmonary arteries, as a consequence of vascular damage associated with viral infection and severe inflammation, rather than emboli from peripheral veins. LE-DVT screening to prevent PE in this setting might be ineffective (26, 27).

Our study has some limitations. First, the sample size is relatively small due to low peak of COVID-19 patients in our ICU during the study period. Therefore, this study has not enough power to detect a difference in mortality between groups. Secondly, LE-DVT screening was based on a single LE-CCUS at 7 ± 2 days. In literature, ultrasound screening protocol vary from one single examination (time from ICU admission not specified) to repeated examinations every 5–7 days (14–16, 18). The results could have been different with earlier screening or repeated LE-CCUS.

In conclusion, a systematic approach with screening for DVT at 7 ± 2 days of ICU admission does not appear to be associated with a higher diagnosis of VTE or a lower cumulative incidence of PE. Further studies are needed to evaluate the value of routine screening for DVT in critically ill patients admitted for COVID-19 pneumonia.

## Data Availability Statement

The datasets analyzed during the current study are available from the corresponding author on reasonable request.

## Ethics Statement

The studies involving human participants were reviewed and approved by Toulouse University Hospital local ethic committee (number's register: 2020-091). Written informed consent for participation was not required for this study in accordance with the national legislation and the institutional requirements.

## Author Contributions

F-XL and FV-B designed the study, performed data collection and analysis, interpreted the data, wrote the manuscript, and gave final approval of the manuscript before submission. VM and AB-R designed the study, interpreted the data, revised the manuscript, and gave final approval of the manuscript before submission. AR interpreted the data, wrote the manuscript, and gave final approval of the manuscript before submission. BC and AT performed data collection, interpreted the data, revised the manuscript, and gave final approval of the manuscript before submission. TG and SS interpreted the data, revised the manuscript, and gave final approval of the manuscript before submission. All authors contributed to the article and approved the submitted version.

## Conflict of Interest

The authors declare that the research was conducted in the absence of any commercial or financial relationships that could be construed as a potential conflict of interest.

## Abbreviations

95% CI: 95% confidence interval
ARDS: Acute respiratory distress syndrome
CCUS: Complete compression venous ultrasonography
COPD: Chronic obstructive pulmonary disease
CTPA: Computed tomography-pulmonary angiography
CT scan: Computed tomography scan
DVT: Deep vein thrombosis
ECMO: Extracorporeal membrane oxygenation
eGFR: Estimated glomerular filtration rate
HR: Hazard ratio
ICU: Intensive care unit
ISTH: International Society of Thrombosis and Haemostasis
LE-DVT: Lower extremity deep vein thrombosis
LMWH: Low-molecular-weight heparin
PE: Pulmonary embolism
RT-PCR: Reverse transcription polymerase chain reaction
SD: Standard deviation
UE-DVT: Upper extremity deep vein thrombosis
UFH: Unfractionated heparin
VTE: Venous thromboembolism.
